# Resilient Care, Sustainable Future: Bridging Climate Action and Patient-Centered Healthcare

**DOI:** 10.12669/pjms.42.(ICON26).15776

**Published:** 2026-04

**Authors:** Syed Ahmer Hamid, Neelum Mansoor

**Affiliations:** 1 Dr. Syed Ahmer Hamid, Pediatric Oncologist and Chair, Organizing Committee ICON2026.; 2 Dr. Neelum Mansoor, Hematologist and Secretary, Organizing Committee ICON2026.

We are honored to welcome the healthcare fraternity of Pakistan to this special supplement, showcasing the original research work by IHHN faculty and released by Pakistan Journal of medical Sciences (PJMS) on the occasion of 8th Biennial Conference (ICON 26) of the Indus Hospital and Health Network (IHHN). ICON has evolved into one of the most prominent multidisciplinary healthcare conferences in Pakistan, attracting a diverse range of healthcare professionals.^1^ The 2026 theme, **“***Resilient Care, Sustainable Future: Bridging Climate Action and Patient-Centered Healthcare,”* is a call for a profound reimagining of our medical landscape, which is at a critical crossroads. As we navigate through the escalating frequency of environmental crises, the urgency for a paradigm shift in how we deliver care has never been more apparent.^2^

Climate change is no longer a distant threat; it is a current medical emergency. From the increasing prevalence of respiratory diseases due to air pollution to the shifting patterns of vector-borne illnesses and the trauma of extreme weather events, the “climate gap” in healthcare is widening. It is one of the most pressing global challenges today, with far-reaching implications for human health and healthcare systems. This impact is particularly pronounced in developing nations where healthcare infrastructure is already strained. Pakistan’s unique vulnerability to environmental challenges necessitates a healthcare model that is not only high-performing but also climate-resilient.^3^

To address this wide spectrum of challenges, multifaceted approaches are required that include developing a sustainable healthcare infrastructure and strengthening regional collaboration and financing models to create climate-resilient, low-carbon health systems.^4^

The theme of ICON aims to synchronize our national health infrastructure with the United Nations Sustainable Development Goals (SDGs), ensuring that as we treat the patients of today, we are not compromising the world of tomorrow.^5^ Our vision is rooted in IHHN’s eight strategic directions, which form the framework for this discourse:

1. *Universal Health Coverage:* Expanding our reach beyond city centers.
2. *People Centered Policies:* Enhancing the “Indus Way” of free, quality care.
3. *Education & Capacity Building:* Training the next generation of climate-aware leaders.
4. *Research & Development:* Driving innovation through local data and evidence.
5. *Multi-Sectoral Approaches:* Integrating environmental health with clinical practice.
6. *Alliances & Partnerships:* Fostering collaboration between public and private sectors.
7. *Technology-Driven Healthcare:* Utilizing digital health to reduce our carbon footprint.
8. *Innovative Solutions:* Exploring sustainable architecture and green hospital waste management.

This conference reflects IHHN’s unwavering dedication to building a robust, sustainable healthcare ecosystem for future generations. 6 Our program is intentionally aligned with the United Nations SDGs, positioning IHHN as a progressive institution committed to local impact through technology-driven and patient-centered care. Throughout the scientific and plenary sessions, we integrate IHHN’s eight strategic directions to ensure our vision is translated into actionable, resilient outcomes. In the context of this conference, **resilience** refers to the ability of our healthcare systems to not only withstand shocks, be they economic, viral, or environmental, but to adapt and thrive. This conference explores several key pillars:

- **Greening Healthcare Infrastructure:** Reducing the carbon footprint of hospitals through renewable energy and sustainable waste management.
- **Decentralized Care Models:** Utilizing primary care networks to ensure continuity of service when central hubs are compromised.
- **Empowering the Patient Voice:** Ensuring that the transition to sustainable practices does not compromise the dignity, accessibility, or quality of care for the individual.
- **Climate-Smart Policy:** Integrating environmental data into clinical decision-making and public health strategies.

The scientific program featured in this conference represents the intellectual rigor and collaborative spirit of researchers, clinicians, and policymakers from across the nation and the globe. They remind us that while the challenges are systemic, the solutions are often born from local innovation and a shared commitment to excellence with compassion.

As we look toward a sustainable future, we must bridge the gap between environmental advocacy and clinical practice. We hope this event will be a resource for evidence-based practice and a source of inspiration for all healthcare professionals committed to building a more resilient world. Our ultimate goal is to set the stage for the next generation and establish a lasting healthcare legacy.
